# Urban Public Spaces as Restorative Environments: The Case of Ljubljana

**DOI:** 10.3390/ijerph20032159

**Published:** 2023-01-25

**Authors:** Katarina Polajnar Horvat, Daniela Ribeiro

**Affiliations:** Research Centre of the Slovenian Academy of Sciences and Arts, Anton Melik Geographical Institute, 1000 Ljubljana, Slovenia

**Keywords:** urban public spaces, green spaces, urban areas, restorative environments, well-being, isolation, sustainability

## Abstract

In this study, we used a survey to examine how urban residents in Ljubljana, Slovenia, value and use distinct urban public spaces. Specifically, we were interested to assess if urban public spaces in the city are used/perceived as restorative environments. To do this, we addressed the question: To what extent do restorative dimensions differ in nine selected urban public spaces, varying in size, design, amenities, number of visitors, and, most importantly, degree of naturalness? Results from survey allowed to determine to what extent the selected urban public spaces in Ljubljana differ in terms of their perceived degree of restoration. We hypothesized that urban public spaces with a higher degree of naturalness in the city have a higher restoration value than urban public spaces with a lower degree of naturalness. Surprisingly, the urban public space that was above average on most restorative dimensions was the Old Town. These results are somewhat at odds with the attentional restoration theory, which states that the combination of dimensions is most typical of natural environments. However, this could be an indicator of the effectiveness of the city’s current policies to improve the quality of life for its citizens.

## 1. Introduction

Most Europeans live in urban or suburban areas, which offer many opportunities, such as access to economic, social, and cultural life, to services such as education and health care facilities, and the opportunity to meet in public spaces. On the other hand, the activities that create and shape urban areas cause negative environmental impacts, such as pollution, space and information congestion that threaten the quality of life of people living there [1,2].

Recent studies have shown that people living in densely populated urban areas are constantly exposed to stimuli that require high levels of attention and can lead to mental fatigue [3]. Coping with the various daily challenges of living in urban environments puts a strain on people’s physical, social, and psychological states, thus directly and indirectly affecting their health [1]. 

Here, the term “restorative” or “restoration” refers to the experience of the psychological and/or physiological recovery process triggered by certain environments and their configurations, e.g., restorative environments, to transform negative states into positive ones [4]. Among the various theories explaining restorative environments, research has been guided by the modified attentional restoration theory [5,6]. This states that people are better able to focus when they spend time in nature or view natural scenes [5,7] and assumes that the environment can counteract the fatigue of focused attention when the relationship between people and the environment is characterized by a number of features: fascination, novelty, escape, extension or connectedness, and compatibility [5,7].

Many studies have shown that natural environments, where we can isolate ourselves from everyday stress and mental fatigue, contribute to relaxation to a greater extent than urban environments [8,9,10]. Spending time in a relaxing natural environment helps reduce stress, promotes positive moods, feelings, and well-being, prevents illness, and facilitates recovery from disease [7,11,12]. 

People have always believed that the natural environment has a positive effect on them, so they wanted it near them. The hanging gardens of Babylon or the greenery in Roman cities testify to a closer contact with nature [13]. Van den Berg and colleagues [14] have shown that just observing nature helps us; people find natural scenes more attractive than urban scenes, and spending time in nature is also very important to many. Patients who recovered in rooms with a view of greenery recovered faster and with less difficulty, students who watched movies about nature recovered from stress faster than those who watched anything else. 

How important nature is for people and the contact with it in the city is shown especially in urban green spaces [15]. People’s relationships with green spaces are inextricably linked to the spatial expansion of cities and are reflected primarily in their responses to the loss of human contact with nature [15]. For this reason, there seems to be a growing awareness of the importance of contact with nature. In addition to their economic function and benefits, such as higher real estate prices [16], or ecological benefits, such as improved air quality or increased resilience to climate change [17], the recreational and leisure functions of urban green spaces are of particular importance. There is ample evidence that spending time in green spaces is associated with an increase in physical activity and a decrease in sedentary behavior, thereby improving the mental well-being and overall health of urban residents [18,19,20,21]. Based on this evidence, the public health benefits of urban green spaces are repeatedly recognized in reports from WHO, which urge increasing access to public open and green spaces with appropriate recreation facilities for all ages to promote active recreation (e.g., [22]).

In our study, we addressed the question of the extent to which the restorative dimensions studied differ in nine selected urban public spaces that vary in size, design, amenities, visitor numbers, and, most importantly, in the varying degree of transformation of the natural environment. Thus, the central aim of the study is to determine to what extent the selected types of public spaces in Ljubljana differ in terms of the perceived degree of restoration in the environment. We hypothesized that less-remodeled public spaces in the city have a higher restorative impact than more remodeled ones.

## 2. Materials and Methods

Due to the diversity of human needs, lifestyles, and leisure habits, people spend their leisure time in different areas that vary in terms of facilities, activity opportunities, and other characteristics, such as the sharing of green space. These are not only areas with a purely recreational function, such as sports fields, children’s playgrounds or urban green spaces, but also those where people meet and spend their leisure time in the broadest sense of the word (e.g., shopping malls, old city center). 

### 2.1. Study Area and Selected Urban Public Spaces

Ljubljana, the capital of Slovenia, has made significant efforts in recent years to develop features that distinguish the city from others and make it an attractive place to live and visit. Features such as the creation of a healthier and greener environment, a distinctive cultural atmosphere, and a vibrant social scene have impacted both residents and the city’s public spaces [23]. Data from the European Environmental Agency shows that Ljubljana appears to be among the cities with the highest proportion of total green space (67%), with the total of green infrastructure making up, on average, 42% of the city area in 38 EEA member countries [24]. 

Ljubljana’s sustainable policy has led to the city being nominated for, and winning, the European Green Capital 2016 award [23]. The award was given to Ljubljana for achieving high environmental standards, setting ambitious goals for further environmental improvement and sustainable development, and acting as a role model for other cities. As part of its ambitions to win the European Green Capital award, Ljubljana has recently paid increased attention to improving the quality of public spaces [25].

**Figure 1:**
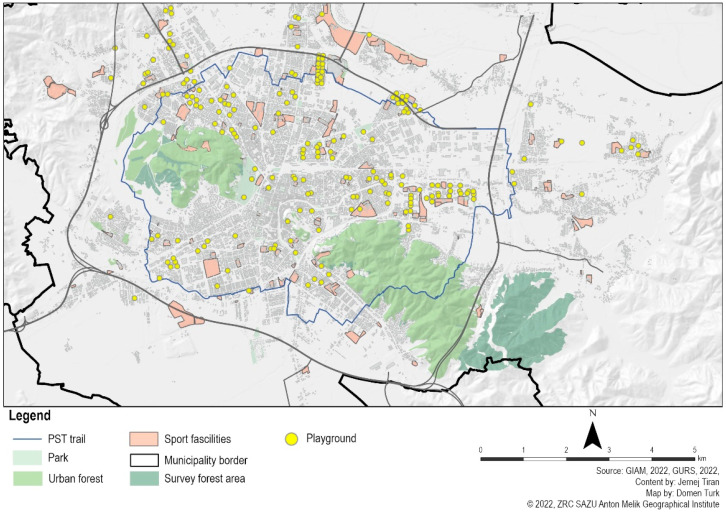
Map of urban public spaces used for recreation in the city of Ljubljana.

Based on available public data, expert opinions, and a typology of urban green spaces [26], we created an inventory, typology, and mapping of urban public spaces in the city of Ljubljana (see Figure 1). In order to compare more than just one type of natural setting against one type of built setting we also included other types of urban public spaces, such as the shopping mall and old city center. Thus, this study assessed the restorative impacts of different types of urban public spaces. We selected the following types of urban public spaces: urban forests, urban parks (small and large), green spaces on the riverbank, playgrounds, sports facilities, neighborhood green spaces, shopping malls, and the city center.

**Figure 2:**
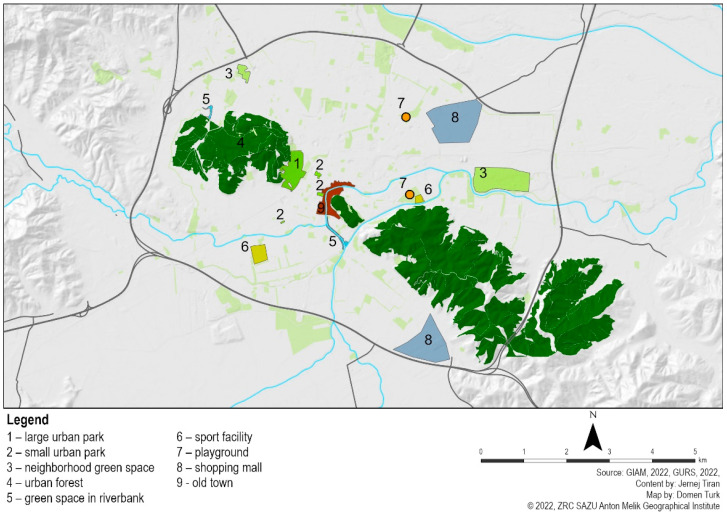
Recreation hot spots in urban public spaces selected for surveying.

In the next step, we identified one or two restoration spaces for each type, which are so-called “hot spots” where people spend most of their leisure time (see Figure 2). We selected representative and frequently visited places in order to achieve a good response rate in the users’ surveys (see Table 1). 

### 2.2. Methods 

The present study used a modified questionnaire based on the perceived restorative scale [27] and the restorative component scale [7], consisting of 15 items (see Table 2), which measured five restorative dimensions: escape, fascination, coherence, compatibility, and novelty, each with three statements. Respondents indicated on a 6-point Likert scale (1–I do not agree at all, 2–I do not agree, 3–I partially disagree, 4–I partially agree, 5–I agree, 6–I completely agree) the extent to which the statement fit their experience in the selected urban public space. The statements were given in random order. We used only statements with positive connotations (see Table 2), as it was shown that the inclusion of mixed positive and negative statements within a single scale can lead to differences that are attributed to the method of measurement rather than the actual differences shown [28,29,30]. Analysis of the statements allowed for the differentiation between urban public spaces varying in restorativeness.

We personally reached out to the widest possible range of visitors at the selected urban public spaces and requested them to report how they experience these environments. Each respondent answered the questions only for the specific public space in which he or she was currently located and which represented the selected type of urban public space. The sample size was 905 people, and the condition for participation in the field survey was that the respondents had lived in Ljubljana for at least one year.

Initially, we intended to have an equal number of surveys for each type of urban public space but in some areas we gathered a higher number of surveys, therefore this number is slightly different among public spaces (see Table 1). The survey included some basic sociodemographic data such as gender, age, and education, in order to achieve a statistically significant sample.

### 2.3. Statistical Analysis

The statistical program SPSS was used to analyze the results. To measure the reliability of the restorative dimensions, we used the Cronbach Alpha test [31], which is the simplest and most commonly used method for assessing the reliability of measurements. It is used to test the association between statements within individual sets of questions and is based on covariance and correlation coefficients between all variables measuring the same construct. It can take values in the interval from 0 to 1. The higher the alpha value, the more reliable the measurement procedure. One speaks of reliability when α is greater than 0.5 [32]. 

We used the t-test to check the association between gender and restorative dimensions. We used the so-called “Independent-Samples T-Test”, which tested whether the mean of the selected variable differed between two groups of units. In this way, we were able to detect differences in the perception of the studied phenomena between men and women. To determine the relationship between numerical and non-numerical variables (age, education), we used the one-way analysis of variance “Oneway Anova”.

## 3. Results and Discussion

### 3.1. Sociodemographic and Exploratory Data Analyses

In terms of ideal sampling, the objective was for each urban public space to provide 100 participants. Discrepancies in the number of completed surveys in certain spaces appeared, as obtaining an exactly the same number of participants for each space was unrealistic. The surveys were conducted using the same recruitment method in each urban public space. 

The survey was completed by 905 respondents, of which 58.2 % were women, 41.3% were men, and 0.5 % did not want to answer the question. Regarding the age structure, most of the respondents are between 25 and 44 years old (39.5%), followed by 45–64 years old (26.5%), 65 years old and older (18.8%) and 15-24 years old (14.9%), and 0.3 % of the respondents did not provide any information. In terms of educational structure, the majority of respondents had a high level of education: 57.1% had a master’s degree, doctorate, post-secondary education, or university degree, while 30.5% had a secondary education, only 4.3% had a vocational education, 7.6% had a primary education, and 0.5% of respondents did not give any answer. 

### 3.2. Survey Results

The reliability of the restorative dimensions studied (escape, fascination, coherence, compatibility, and novelty), was evaluated using the Cronbach alpha coefficient. The analyses performed with the SPSS program showed that all five dimensions have a high level of reliability and form quality components (see Table 3).

People in the urban public spaces studied valued compatibility the most, with an average score of 4.39. Compatibility reflects the feeling of enjoyment and agreement in a given environment. It is higher when engaged in an activity with which one is familiar. To be compatible with an environment, it must be one in which the person spends time based on intrinsic motivation and personal preferences [33]. This was followed by coherence with an average score of 4.19. This dimension refers to the quality of a restful environment that encourages full engagement and involvement [34]. It means that the environment has no unusual or unexpected features and that one feels comfortable and relaxed in the selected environments. The third highest rated dimension was novelty with an average score of 4.14. Novelty refers to the unexpected and surprising [35]. It means that the environment is new to someone or different from their daily environment [36]. The next dimension was escape (4.04), which refers to the feeling of escaping from one’s usual thoughts and worries and distracting oneself from the environment that occupies one’s attention and energy [33]. The least appreciated dimension was fascination (4.01), where attention is held without any effort. 

According to the results showed on Table 4, people had the greatest sense of escape in the urban forest (4.73), which is not surprising considering that research shows that the urban forest is the most different from other urban environments in terms of stimuli, and therefore is the environment where people can forget about everyday urban life [37,38]. Interestingly, respondents mentioned sports facilities as the second place where they experienced escape (4.61). The reason for this is that sport in itself is a distinctly relaxing activity where one can switch off the mind [39]. On the other hand, there is an increasing importance of leisure and sports. Neighborhood green spaces were the third place where people experienced an escape (4.35). In this case, the reason could be the desire to escape from a small and cramped flat, which is typical especially for large flat blocks with small apartments. On the other hand, the respondents felt the least sense of escape in the shopping center (3.03). Shopping malls involve a large covered space, which in itself provides a variety of stimuli that cause additional arousal in a person. 

The feeling of fascination was felt most strongly by respondents in the Old Town (4.48) and by riverbanks (4.26). This is not surprising, as these are very rich points in terms of cultural (Old Town), aesthetic (Old Town and surrounding area), and biotic (riverbank) values, which could be called “heritage hotspots” in the case of the Old Town and “nature hotspots” in the case of the riverbank area. Respondents felt the least fascination for small city parks (3.63) and neighborhood green spaces (3.82). The reason for this is that respondents are very familiar with these two environments, as they are in close proximity to their homes, and these are frequently visited. They felt the strongest sense of coherence in the Old Town (4.53), sports facilities (4.48) and children’s playgrounds (4.42), and the least in small city parks (3.72) and urban forests (3.76). The greatest sense of compatibility was characteristic of sports facilities (4.86) and large urban parks (4.78), while the least sense was attributed to shopping malls (3.64) and small urban parks (3.97). Nowadays, sports are an increasingly important part of leisure time [40], and people increasingly identify and feel fulfilled by sports activities. Moreover, they feel compatible with a large urban park, which is, among other things, a space for people to engage in sporting activities such as running, outdoor fitness, yoga, basketball, etc. On the other hand, it is not surprising that they feel least compatible with a shopping mall, as it is an artificial space with many stressful stimuli. Respondents felt most surprised in the Old Town (4.56) and in the urban forest (4.40), and least surprised in green spaces in the neighborhood (3.59) and in small urban parks (3.79). The reason for this is that the appearance of the Old Town is constantly changing due to various events, everyday hustle and bustle, the changing appearance of service infrastructure, etc. The urban forest is also constantly changing and nature shapes its dynamics (such as seasonal changes). In addition, the respondents visit the urban forest on average less frequently than other urban public spaces. On the other hand, we are not surprised by the answers concerning the least feeling of novelty in neighborhood green spaces and small urban parks, since these areas are very frequently visited by the respondents.

Using the independent samples *t*-test, we found that participants’ responses to all five restorative dimensions were not statistically significantly different with respect to their gender, age, and educational structure, but that there were some interesting differences between participant groups (see Table 5). In terms of gender structure, females rated higher on all the restorative dimensions examined. However, both genders rated compatibility the highest, followed by coherence, novelty, escape and fascination.

In terms of age structure, it was the more mature respondents, aged 45 to 64, who valued restorative components to a greater extent (see Table 6). The reason for this could be that the more mature age group is the most exposed to stress. On the one hand, this is the time when the highest work activity takes place from the point of view of career, raising children and caring for parents. On the other hand, this is also the time when health problems begin. 

As for the educational structure, the results did not reveal any significant differences between the respondents.

## 4. Conclusions

Understanding the restorative dimensions and their effects provided by urban public spaces, especially in Ljubljana, is a relatively new endeavor. The aim of this research was to determine the extent to which the selected urban public spaces differed in terms of the degree of environmental restoration perceived by people. The results of the survey showed that the differences between the different types of urban public spaces in terms of the perceived restorative dimensions are generally smaller than one would expect. The Old Town scored the highest overall and was above average for most restorative dimensions. It appears that the Old Town of Ljubljana is a well-designed area where all the dimensions examined–escape, fascination, coherence, compatibility and novelty–are well represented. This could be an indicator of the effectiveness of the city’s current policies to improve the quality of life for all citizens, as access to public spaces for all and the inclusion of all people in public activities are important features of sustainable development that the city strives for [23]. 

The second highest score in all dimensions were the sports facilities. One of the reasons for this could be that they were built as multifunctional places and are well designed from an urban planning perspective. These results are somewhat at odds with the attentional restoration theory, which states that the combination of dimensions is most typical of natural environments.

Large urban parks and green spaces on riverbanks were also rated as above average in most dimensions. Urban dwellers seem to prefer well-kept, organized, and tidy places, even if these places are only an artificial reflection of nature. The presence of water seems to have an additional positive influence on people’s ratings. A considerable part of the Old Town, which scored the highest in terms of restorative dimensions, includes water–the river Ljubljanica, where people spend their time in various ways, reading, walking, running, etc. This positive contribution of naturalness to the perceived restorativeness corroborates with a Swedish research study, which revealed that green spaces exemplifying certain dimensions were preferred by stressed individuals [41]. Urban forests topped the list of escape options. One of the reasons for this could be that they support many different types of activities and the associated disconnection from everyday life. On the other hand, urban forests did not score as well on other restorative dimensions, such as fascination and coherence. This fact contrasts with results in the literature, which consider urban forests as the type with the highest value of restorativeness [42,43]. Moreover, there is a body of evidence [44,45] suggesting that the presence of very dense vegetation may compromise restoration by evoking feelings of insecurity.

Shopping malls performed quite poorly on the restorative dimensions studied, which severely limits the potential of shopping as a restorative environment. On the other hand, promoting restoration in historic districts seems to make sense. Even the restorative value of a historic district is considered rather low by other studies; such places offer other benefits and characteristics, especially if, as is the case of Ljubljana, they are designed for people rather than motorized traffic. 

Even though noise exposure and other types of pollution in urban areas cause important issues for modern societies [46], this was not specifically addressed in this study. Analysis of the soundscape, for instance, could be a good method to identify urban outdoor spaces with a high value of restorativeness, as was similarly done by other studies [47].

In addition, the promotion of restorative environments should be strengthened [48], especially since the health benefits of physical activity and relaxation from stress have not yet been explicitly addressed in the Urban Agenda for the EU. We hope that the results presented will contribute to the understanding and recognition of the urban public spaces as useful restorative environments for restoration from ‘mental fatigue’ or the depletion of cognitive resources [5,6]. It is hoped that this type of research will also help planners make the inclusion of spaces that are most effective in providing restoration in cities a priority.

## Figures and Tables

**Table 1:** Typology of urban public spaces and numbers of completed surveys per public space.

	Type of Urban Public Space	Number of Completed Surveys
1	large urban park	101
2	small urban park	100
3	neighbourhood green space	100
4	urban forest	101
5	green spaces on riverbank	100
6	sport facility	100
7	playground	101
8	shopping mall	101
9	old town	101

**Table 2:** Perceived restorativeness items used in the present study, grouped according to the five restorative dimensions.

Dimensions	Survey Items
Escape	I can forget about my daily responsibilities in/on ______.
	In/on ______ I feel like running away from everything.
	In /on ______ I relax and get rid of negative thoughts.
Fascination	I can see many beautiful and interesting things in /at ______.
	______ makes me curious about many things.
	I can discover a lot and do a research in/on ______.
Coherence	I can easily see how things are organized in/on ______.
	Everything I see in/on ______ matches with this space.
	______ is nicely arranged.
Compatibility	What I can see and do in/on ______ matches my expectations.
	I can do the things I like in/at ______.
	I feel like I belong here.
Novelty	______ is quite different from my everyday environment.
	I do things in/on ______ that are different from my everyday activities.
	I find ______ unique.

**Table 3:** Reliability of restorative dimensions measured with Crombach Alpha (σ).

Restorative Dimension	Mean Value	σ
Escape	4.04	0.90
Fascination	4.01	0.87
Coherence	4.19	0.77
Compatibility	4.39	0.80
Novelty	4.14	0.70

**Table 4:** Extent to which the restorative dimensions differ in 9 selected urban public spaces.

Restorative Dimension		Escape		Fascination		Coherence		Compatibility		Novelty	
Type of Urban Public Space	N	Mean Value	St. Dev.	Mean Value	St. Dev.	Mean Value	St. Dev.	Mean Value	St. Dev.	Mean Value	St. Dev.
Large Urban Park	101	4.46	1.03	4.03	1.99	4.35	1.67	4.78	1.97	4.17	1.04
Small Urban Park	100	3.40	1.25	3.63	1.01	3.72	0.95	3.97	1.02	3.79	1.12
Neighbourhood Green Space	100	4.35	1.32	3.82	1.33	4.27	1.15	4.13	1.89	3.59	1.71
Urban Forest	101	**4.73**	1.98	3.83	1.84	3.76	1.82	4.72	2.23	4.40	2.30
Green Space on Riverbank	100	3.98	1.10	4.26	0.84	4.16	0.70	4.52	6.88	4.17	0.95
Sport Facility	100	4.61	1.30	3.87	2.03	4.48	1.92	**4.86**	1.93	4.09	1.14
Playground	101	3.96	1.23	4.15	1.25	4.42	1.08	4.41	1.07	4.20	1.22
Shopping Mall	101	3.03	1.88	3.98	1.98	4.06	1.12	3.64	2.02	4.33	1.10
Old Town	101	3.89	1.14	**4.48**	0.80	**4.53**	0.71	4.51	0.79	**4.56**	1.01
TOTAL	905	4.04	1.36	4.01	1.45	4.19	1.24	4.39	2.20	4.14	1.29

Note: The bold numbers correspond to the highest mean values.

**Table 5:** Association between gender and restorative dimensions.

Restorative Dimension		Escape		Fascination		Coherence		Compatibility		Novelty	
Gender	N	Mean Value	St. Dev.	Mean Value	St. Dev.	Mean Value	St. Dev.	Mean Value	St. Dev.	Mean Value	St. Dev.
female	529	4.15	1.42	4.12	1.55	4.27	1.27	4.43	3.21	4.21	1.32
male	376	3.93	1.29	3.90	1.34	4.10	1.21	4.34	1.19	4.07	1.25
Total	905	4.05	1.36	4.01	1.45	4.19	1.24	4.39	2.20	4.14	1.29

**Table 6:** Relationship between age structure and restorative dimensions, using the “Oneway Anova”.

Restorative Dimension		Escape		Fascination		Coherence		Compatibility		Novelty	
Age Structure	N	Mean Value	St. Dev.	Mean Value	St. Dev.	Mean Value	St. Dev.	Mean Value	St. Dev.	Mean Value	St. Dev.
15-24 years old	135	4.02	1.22	3.84	0.90	4.20	0.88	4.21	5.99	4.02	1.10
25-44 years old	358	4.07	1.27	3.93	1.12	4.15	0.96	4.46	1.08	4.11	1.08
45-64 years old	240	**4.10**	1.30	**4.17**	1.11	**4.24**	1.00	**4.58**	1.03	**4.27**	1.09
65 years old and older	170	3.96	1.31	4.11	1.20	4.18	0.97	4.31	1.08	4.16	1.10
Total	905	4.04	1.30	4.01	1.13	4.21	0.99	4.39	2.51	4.14	1.11

Note: The bold numbers correspond to the highest mean values.

## Data Availability

Not applicable.
